# Molecular Mechanisms of Specific Cellular DNA Damage Response and Repair Induced by the Mixed Radiation Field During Boron Neutron Capture Therapy

**DOI:** 10.3389/fonc.2021.676575

**Published:** 2021-05-19

**Authors:** Kamila Maliszewska-Olejniczak, Damian Kaniowski, Martyna Araszkiewicz, Katarzyna Tymińska, Agnieszka Korgul

**Affiliations:** ^1^ Department of Physics and Biophysics, Institute of Biology, Warsaw University of Life Sciences-SGGW, Warsaw, Poland; ^2^ Centre of Molecular and Macromolecular Studies, Polish Academy of Sciences, Lodz, Poland; ^3^ Faculty of Physics, University of Warsaw, Warsaw, Poland; ^4^ Nuclear Facilities Operations Department, National Centre for Nuclear Research, Otwock, Poland

**Keywords:** neutron mixed-beam, BNCT (boron neutron capture therapy), DNA damage, DNA repair, high-LET, low-LET radiation, complex DNA damage

## Abstract

The impact of a mixed neutron-gamma beam on the activation of DNA damage response (DDR) proteins and non-coding RNAs (ncRNAs) is poorly understood. Ionizing radiation is characterized by its biological effectiveness and is related to linear energy transfer (LET). Neutron-gamma mixed beam used in boron neutron capture therapy (BNCT) can induce another type of DNA damage such as clustered DNA or multiple damaged sites, as indicated for high LET particles, such as alpha particles, carbon ions, and protons. We speculate that after exposure to a mixed radiation field, the repair capacity might reduce, leading to unrepaired complex DNA damage for a long period and may promote genome instability and cell death. This review will focus on the poorly studied impact of neutron-gamma mixed beams with an emphasis on DNA damage and molecular mechanisms of repair. In case of BNCT, it is not clear which repair pathway is involved, and recent experimental work will be presented. Further understanding of BNCT-induced DDR mechanisms may lead to improved therapeutic efficiency against different tumors.

## Introduction

Boron neutron capture therapy (BNCT) is a radiation therapy that can selectively target neoplastic tissue with an advantage over conventional radiotherapies. BNCT is a binary approach in which boron-10 (^10^B)-labeled compounds such as low molecular weight boron-containing drugs, boronophenylalanine (BPA) or sodium borocaptate (BSH), are administered before irradiation with thermal or epithermal neutrons (1–4). BNCT is effective in treating high-grade gliomas, recurrent head and neck tumors, and cutaneous and extra-cutaneous melanomas (1, 2). ^10^B-enriched compounds deliver high concentrations of ^10^B to the target tumor cells, followed by thermal neutron or epithermal neutron irradiation, thermalized inside tissues. Research began in the 1930s, shortly after Chadwick discovered neutron in 1932, when Taylor and Goldhaber described the (^10^B(n, α)^7^ Li) capture reaction in 1935 (5). However, the idea of exploiting the neutron capture reaction in cancer therapy was put forward by Gordon Locher in 1936. This concept assumes that the interaction of thermal neutrons (<0.4 eV) with tissue deposit a radiation dose that can be kept under tolearnce levels. The essence of this therapy is the interaction between ^10^B and thermal neutrons which is sufficient to kill the tumor cells (2):

To obtain the desired results, an optimal amount of ^10^B must be selectively delivered to all cancer cells (20 µg/g weight or ~10^9^ atoms/cell) and an optimal fluence of thermal neutrons should be absorbed to obtain a lethal effect using the (^10^B(n, α)^7^ Li) capture reaction (6). The α (^4^He) particles and lithium (^7^Li) nuclei released from the neutron capture reaction (^10^B(n, α)^7^Li) are short-ranged (5–9 µm), making the ^10^B distribution critical for BNCT, thus limiting the damage to the cells containing only ^10^B (7). If the boron compounds are selectively delivered to tumor cells and accumulate there, BNCT meets the premise that this therapy selectively destroys only tumor cells (1). Therefore, the biological effect of this therapy depends critically on the gross and microscopic distributions of boron in tissues.

Neutrons undergo a great variety of nuclear reactions in biological targets, thus producing a mixed field of secondary particles, and nuclear reaction cross-sections are strongly dependent on neutron energy (8). The mixed radiation field consists of a mixture of components with different linear energy transfer (LET) characteristics that act independently (3). Epithermal neutrons (0.4 eV < E_epi_ < 10 keV); penetrating tissue are reduced to the thermal energy range (< 0.4 eV) as a consequence of collisions with atoms of hydrogen and captured by the ^10^B nucleus. Released α-particles and ^7^Li nuclei have high LET. The interaction of the neutron beam with the nuclei of elements in tissue causes a nonspecific background, consisting of a mixture of high- and low-LET components, to appear. Low-LET γ-rays are released due to thermal neutron capture by hydrogen in the (^1^H(n, γ)^2^H) reaction, whereas high-LET protons are released after the capture of thermal neutrons by nitrogen in the tissue by the (^14^N(n,p)^14^C) reaction. High-LET recoil protons also appear through collisions with hydrogen nuclei (^1^H(n, n’)p) reaction in tissues and are produced by fast neutrons (>10 keV) in the neutron beam. γ-rays come from the beam infrastructure, reactor core, and beam shaping assembly. Therefore, in BNCT, mixed radiation of primary and secondary particles of various energies are involved. Undoubtedly, BNCT involves mixed-field irradiation (7). Early trials of BNCT were limited in their ability to estimate and predict responses to a complex, mixed radiation field (7).

It was demonstrated that DNA damage increases with LET of radiation (1). Recent studies have reported that radiation with high LET is also observed in proton and carbon ion therapy, and is more effective than low-LET radiation such as in X-rays or γ-rays (9). The higher the LET, the higher is the relative biological effectiveness (RBE). This enhanced RBE is determined by a unique type of DNA damage, characterized by clustered, complex lesions that override DNA repair capacity in tumor cells. These sites are two or more lesions, in close proximity (within 1 to 2 helical turns of DNA) owing to radiation, and are integrated into complex DNA-double stranded breaks (DSBs) (10, 11).

In this study, we aimed to describe the molecular mechanisms of cellular response to DNA damage, DNA damage response (DDR) induced by BNCT with an emphasis on mixed field radiation, and effects of low and high LET radiation in different cancer cell lines. The impact of the mixed beam on the activation of DDR proteins and repair pathways is poorly understood, especially involving BNCT (9, 11–13).

## DNA Damage Response and Repair Pathways After the Mixed Radiation Fields

**Figure 1:**
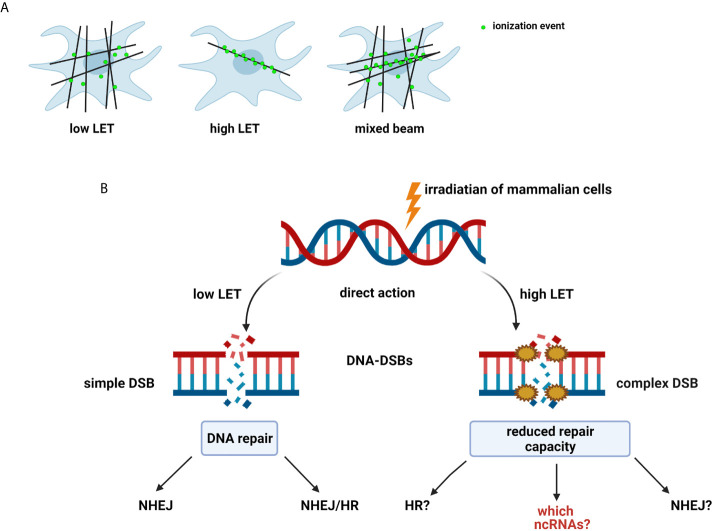
**(A)** Types and effect of radiation according to linear energy transfer (LET): Low-LET radiation produces sparse ionization along its track, homogeneously within a cell. High-LET radiation causes dense ionization along its track. Mixed beam— both effects observed within a single cell (11, 12, 16, 17). **(B)** Radiation-induced DNA damage: DSBs induced by low-LET radiation are repaired by non-homologous end-joining pathway (NHEJ) alone or NHEJ and homologous recombination (HR). Mechanisms of repair of complex DSBs induced by high-LET radiation are not fully determined (9). Created with BioRender.com.

The effect of radiation on cells can be described as a double action. Ionizing radiation (IR) has anticancer effects by inducing DNA damage in proliferating cancer cells or injuring healthy cells (14). IR most commonly causes DSBs, the most genotoxic DNA lesions, leading to cell death or mutations that can be repaired *via* specific repair pathways. DNA repair is one of the most critical processes essential to genome integrity, maintaining all cellular functions and survival (15). IR is characterized by its RBE and is related to the LET by depositing energy in specifically structured tracks (9). High-LET radiation produce denser ionization than low-LET radiation, which is sparsely ionizing (**Figure 1A**). Neutrons are high-LET and can induce complex DNA damage, like other high LET particles (alpha particles, carbon ions, protons), while γ-rays is low-LET radiation (9, 11). The effects of both low- and high-LET radiation are observed within a single cell in the case of mixed beams (**Figure 1A**). Interestingly, during their lifetime, humans are exposed to mixed fields of low- and high-LET radiation, like during plane and space flights (exposure to neutrons, γ-rays, and protons), in closed spaces and areas high in alpha-emitting radon-222, and alpha and γ-emitting radium-226. Cancer patients are exposed to a mixed field during radiation therapy (18). Many cancer therapies besides BNCT, such as intensity-modulated radiotherapy (IMRT), proton therapy, and hadron therapy produce mixed fields of radiation (12, 13, 19, 20), and recent studies have focused on the measurement of secondary γ-rays (prompt-γ production) emitted during proton beam and carbon ion irradiation (21, 22).

**Figure 2:**
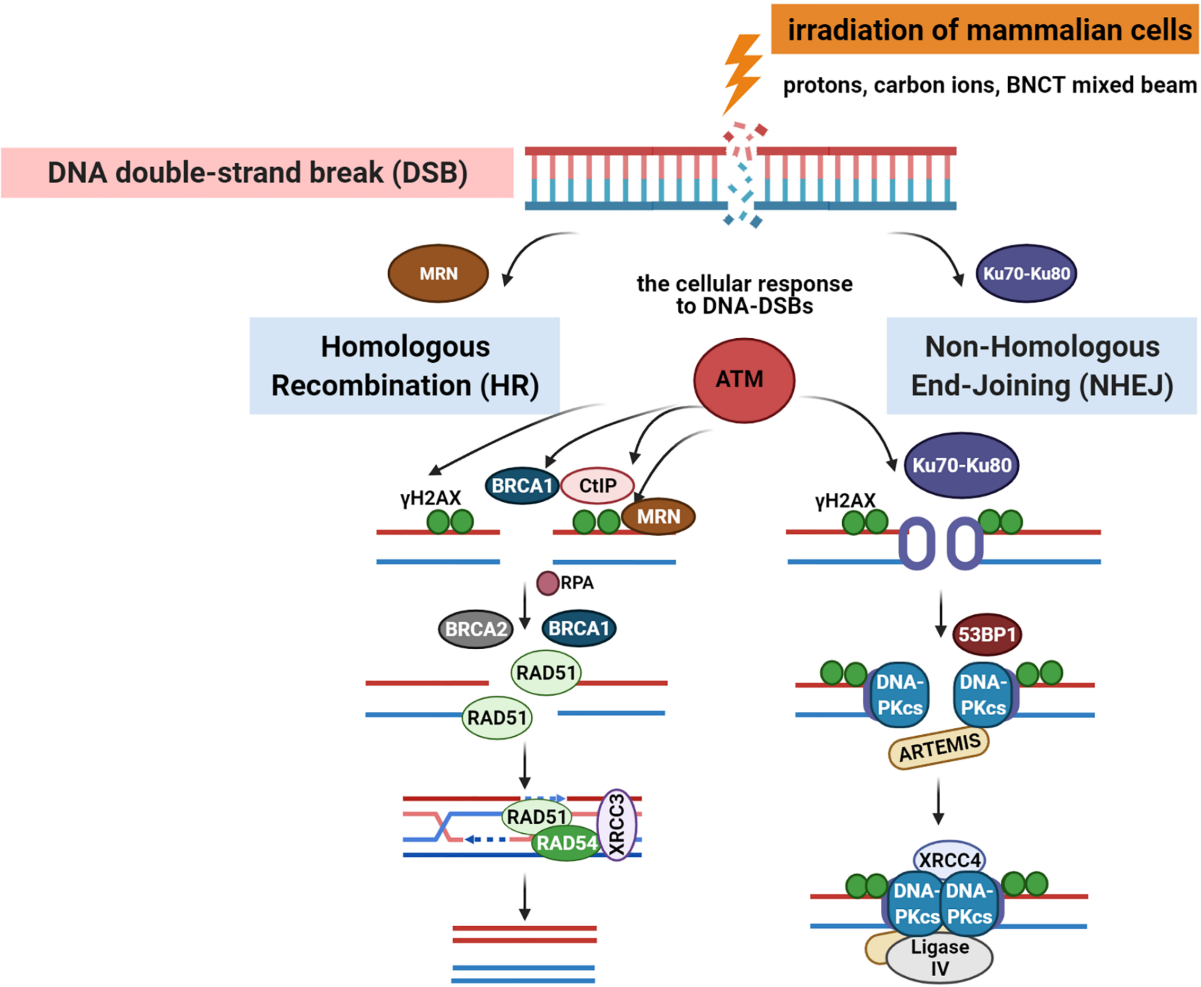
DNA**-**double-stranded breaks repair pathways: homologous recombination repair (HRR) and non-homologous end-joining (NHEJ) pathway induced by low and high linear energy transfer (LET) radiation (15, 23). Created with BioRender.com.

Radiation-induced DNA-DSBs in higher eukaryotic cells are repaired either by the non-homologous end-joining pathway (NHEJ) or homologous recombination repair (HRR) (**Figure 2**). Phosphorylation of histone γ-H2AX (a variant of the H2A protein family) at serine-139 by ATM (Ataxia telangiectasia mutated kinase) belonging to the PI3 (phosphatidylinositol-3) kinase family is the initial step of DSB induction (24, 25). γ-H2AX is dephosphorylated when DNA repair is completed; therefore, the DSB marker γ-H2AX is studied extensively through the characterization of foci formation, size, and quantity. Ionizing radiation-induced foci (IRIF) are brighter and larger after high-LET exposure compared with low-LET radiation (9, 18, 26). The formation of γ-H2AX foci leads to the recruitment and accumulation of DNA damage response (DDR) proteins and chromatin-modifying factors, such as 53BP1 (P53 binding protein 1), MDC1 (mediator of DNA damage checkpoint), BRCA1 (Breast Cancer 1 protein), Mre11/Rad50/Nbs1, PARP-1 (poly(ADP-ribose) polymerase 1), and many others, thus forming radiation-induced foci and co-localization with γ-H2AX through direct or indirect binding (10, 11, 27). 53BP1 is a transcriptional coactivator of the P53 tumor suppressor and acts as an early participant in the cellular response to DNA-DSBs (28). P53 is a transcription factor closely associated with radiation-induced damage response in cells (14). P53 plays a key role in regulating the cell cycle checkpoint and modulating the base excision repair (29). Moreover, it repairs IR-induced DNA damage *via* direct protein-protein interactions with ATM or indirectly by regulating the transcription of genes responding to P53 signaling. The NHEJ pathway involves the Ku70/80 heterodimer, which binds to DNA ends after DSB appearance. This leads to the recruitment of DNA-PK catalytic subunit (DNA-PKcs) to the DSBs, phosphorylation of DNA-PKcs, Ku70/80 heterodimer, and proteins involved in the regulation of the cell cycle. The next step involves Ku70/80 binding to the ends of DSBs, resulting in open access to Ligase IV-XRCC4 (X-ray cross-complementing gene 4) complex. In the HRR mechanism, the Rad52 epistasis gene family is involved, and Rad51 and Rad54 are the key human recombination factors involved in repair mechanisms related to DNA breaks in eukaryotes (30). Rad51 acts by binding to single-stranded DNA (ssDNA) and promotes a search for homolog and DNA strand exchange, while Rad54 activates the pairing function of Rad51 (31). Downregulation of HRR pathway was found to favor error-prone NHEJ pathway machinery, highlighting the significance of HRR repair in genome stability.

The repair of mixed radiation-induced complex DNA damage is poorly understood (9, 12). The mechanism defining which repair pathway is selected is not clear; however, the cell cycle or an inducing factor may be responsible (9, 32, 33). NHEJ acts mainly in G1 and early S phase, with no need for an undamaged DNA template to operate, while HRR operates in the S phase using sister chromatid as the template in a rather error-free manner (10). DSBs that occur in the late S or G2 phase of the cell cycle are repaired by any of the pathways. High-resolution microscopy and real-time imaging show that simple DSBs are quickly repaired by proteins belonging to NHEJ, except DNA-PKcs (10). Interestingly, complex DSBs are slowly repaired, and DNA-PKcs is only recruited to longer-lived complex DSBs (34, 35). DSBs induced by high LET were repaired by NHEJ slowly because of clustered DNA damage (**Figure 1B**). Recent reports have focused on the role of NHEJ in the repair of carbon ion-induced and BNCT-induced damage (9, 35). Moreover, NHEJ has been shown to play a crucial role in DSB repair induced by both clinical proton and carbon ion beams (36). Additionally, NHEJ-deficient cells are more resistant to high LET radiation, relying only on HRR. It was also proposed that complex DNA damage induced by high LET irradiation from high-energy iron ions is repaired by homologous recombination (HR) and not by NHEJ in mammalian cells (37).

The reduced repair capacity after high-LET radiation keeps DNA damage unrepaired for a long time, leading to genome instability and cell death (9, 12, 16). This could be explained by the inability of the DNA repair machinery to relax the chromatin to repair these breaks. Thus, clustering of DSBs after high-LET radiation makes DNA repair more challenging. The health risks of exposure to mixed fields have not been fully determined. It was proposed that the effects of a mixed field of alpha and X-rays exposure could be higher than the additive effects of single beam components (12). Alpha particles and X-rays together produce micronuclei and chromosomal aberrations in peripheral blood lymphocytes (PBLs), above the level assumed by the additive effects of both types of radiation (38, 39). The authors have demonstrated that alpha particles and X-rays interact to produce DNA damage greater than predicted and that DDR is delayed. Moreover, the highest levels of DDR proteins, ATM, P53, and DNA-PK, were observed in cells exposed to the mixed field. Based on the obtained results, the future application of the combination of high and low LET radiation in radiotherapy was proposed. However, more thorough research is needed with high-LET radiation and mixed beams concerning repair dynamics of clustered DNA damage for the application of cancer radiotherapies.

## The Underestimated Role of Small ncRNAs in DNA Damage Response and Repair

RNA transcripts and DDR proteins are known to interact functionally (40). Many recent studies have reported the pivotal role of ncRNAs in DNA repair and genomic rearrangements in different research models (41–44). There is growing evidence that ncRNAs regulate the DDR, especially small microRNAs (miRNAs), which are induced at DNA-DSBs, thus mediating repair (23, 41, 45). Regulatory short miRNAs are ncRNAs encoded in intronic regions of protein-coding genes or in the intergenic regions of the genome (23, 42). Small ncRNAs generated at DNA-DSBs, critical for DDR activation, are termed DDR small RNAs (DDRNAs) and are described in details in a review by Rzeszutek et al. (41). It is considered that some small ncRNAs can regulate the expression of genes encoding DDR proteins, especially those involved in DSB repair; however, the mechanisms are not fully understood (45). DDRNAs are specifically localized to damaged homologous genomic sites in a transcription-dependent manner. Following DNA damage, RNA polymerase II binds to the MRE11/RAD50/NBS1 complex, recruits it to DNA-DSBs, and synthesizes damage-induced long ncRNAs (dilncRNAs) (41, 43). Both dilncRNAs and DDRNAs are involved in DDR focus formation and are associated with 53BP1. For example, overexpression of miR-34c-5p, from the miR-34s family, suppresses Rad51 and upregulates γ-H2AX. These findings highlight a novel mechanism of HR pathway regulation through miRNAs (45). The correlation between miRNAs and DDR is supported by the direct role of mediator proteins ATM and BRCA1 in the synthesis of specific miRNAs (23). Moreover, a variety of studies have shown that miRNAs regulate ATM and DNA-PK. ATM is a target of miRNA-421, miRNA-18a, miRNA-101, and miRNA-100 (23, 46–48). Interestingly, miRNA-101 also suppresses DNA-PKcs in the NHEJ pathway in *in vitro* and *in vivo* cancer models, significantly changing the radiosensitivity of tumors (48). In HR pathway, other miRNAs play an important role in DSB repair: miR-138 targeting H2A.X in osteosarcoma cells, miR-146a and miR-146b-5p targeting BRCA1 in breast cancer, miR-1 targeting BRCA1 in prostate tumor cell lines, and miR-1245 targeting BRCA2 in breast cancer cell lines (23). To attain a deeper understanding of the cellular response to DNA damage, we need a thorough understanding of how DNA damage regulates miRNA expression and how miRNAs affect DDR. This raises the possibility that crosstalk between miRNAs and DDR can efficiently repair DNA and maintain genomic stability. It has been proposed that miRNAs are key regulators for the correct choice among DNA-DSB repair pathways and for repair itself. miRNAs could be useful prognostic markers and miRNA-based therapies could improve the sensitivity of tumor cells to different radiotherapies (49).

## The Impact of BNCT Mixed Radiation Field on DNA Damage Response and Repair

Recently published data have reported that BNCT induces tumor type-specific DNA damage and repair pathways. BNCT has greater potential than conventional photon radiotherapy in cancer treatment, especially in aggressive tumors, and destroys cancer cells with limited effect on healthy cells. However, little is known about the effects of DNA damage induced by a mixed radiation field, such as that used for BNCT (13, 35, 50). However, effects of neutrons have been tested in processes such as DNA damage, apoptosis, chromosomal aberrations, and cell viability. Kondo et al. have shown that DNA damage induced by BNCT is partially repaired by a key player of the NHEJ pathway, ligase IV (35). The authors analyzed the sensitivity of the mouse embryonic fibroblast cell lines *Lig4*−*/*− *p53*−*/*− and *Lig4*+*/*+ *p53*−*/*− to irradiation using a thermal neutron beam in the presence or absence of BPA. It was demonstrated that the *Lig4*−*/*− *p53*−*/*− cell line was more sensitive than the *Lig4*+*/*+ *p53*−*/*− cell line to irradiation with only the beam or with beam and BPA. Another study performed by the same research group using an *in vivo* mouse model after BNCT showed that the DSBs induced by the (^10^B(n, α)^7^Li) reaction were more difficult to repair and stayed longer than γ-rays, suggesting that BNCT has a stronger effect than conventional X-ray or γ-ray radiotherapy (51). The desirable anti-tumor effect of BNCT may be due to the unrepaired DSBs induced by the (^10^B(n,α)^7^Li) reaction. Kinashi et al. presented a study using an *in vitro* model, Chinese hamster ovary CHO-K1 cells deficient in Ku80 protein belonging to the NHEJ pathway (xrs-5 cells), which showed sensitivity to IR during BNCT (52). The DNA-DSBs induced by BNCT were not fully repaired in xrs-5 cells with a high cytotoxicity, and irradiated cells were found to have a reduced DSB repair capacity. Another study on the human thyroid follicular cancer cell line (WRO) demonstrated that HRR is the main activated pathway based on high expression of Rad51 and Rad54 after BNCT (50). The results were different in the human melanoma cell line (Mel J) where both pathways, NHEJ and HRR, were activated after BNCT irradiation. An additional study of DDR after boric acid-mediated BNCT in hepatocellular carcinoma showed inhibition of the growth of Huh7 human HCC cells by induction of DNA-DSBs and apoptosis (53). The authors suggest that HCC cells may undergo G2/M cell-cycle arrest and use the HR pathway to repair BNCT-induced DNA-DSBs.

There is growing evidence and correlation between the tumor suppressor P53 status and the cytotoxic effect of high-LET beams; however, limited studies have been conducted on BNCT (1, 54). *P53* is mutated in some glioblastoma cells, but it is unclear if this mutation affects cellular sensitivity to neutron irradiation. The role of *P53* mutation in the effect of BNCT was tested on several cell lines, including oral squamous cell carcinoma (SCC), and the results obtained by Fujita et al. indicated that mutant-type SCC cells are more resistant than cells with wild-type *P53* due to the lack of G1 arrest and related apoptosis (1). These cells were tested using different methodologies: colony formation assays, proliferation and cell cycle analysis, and expression of cell cycle-associated proteins. Interestingly, the combination of BNCT with adenoviral-mediated gene therapy to introduce the wild-type *P53* gene enhances radiation sensitivity of cells and the effectiveness of BNCT. Another study was performed by Kinashi et al. using glioblastoma cell lines T98G (*P53*-mutant) and A172 (*P53*-wild type) to investigate the relationship between *P53* mutations and sensitivity in combination with the DNA-alkylating agent temozolomide (TMZ) and neutron radiation (54). T98G cells were more resistant to TMZ than A172 cells, and T98G cells were more resistant to neutron irradiation when BPA was administered.

Interestingly, there is scientific evidence that the epidermal growth factor receptor (EGFR) modulates DNA repair after radiation-induced damage by associating with the catalytic subunit of DNA protein kinase (55). It was noticed that cells with certain *EGFR* gene mutations and different levels of EGFR in cancer cells may make the cells differently sensitive to low or high LET radiation because radiation differentially affects tumors and healthy cells (56). In some cases, an increase in the amount of EGFR in cancer was observed after radiotherapy. The invasiveness of neoplastic cells after radiotherapy increased relative to that of the control cells. Overexpression of *EGFR* and *P53* mutations have been linked to treatment resistance in head and neck cancers, including squamous cell carcinoma (HNSCC). *EGFR* is overexpressed in 90% of HNSCCs, and *P53* is the most common somatic mutation. Both EGFR and P53 are implicated in the repair of radiation-induced DNA damage by forming an EGFR–DNA-PK complex. Additionally, EGFR is present on the plasma membrane and upon radiation, evades degradation, and translocates to the nucleus and cellular organelles that generate resistance in cancer cells (57). The results indicate that the dual inhibition of EGFR and HER2 (human epidermal growth factor receptor 2) by afatinib, used for the treatment of non-small cell lung carcinoma (NSCLC), makes cells sensitive to radiation and reduces cell invasiveness. Afatinib is an anilino-quinazoline derivative and inhibitor of the receptor tyrosine kinase (RTK) epidermal growth factor receptor (ErbB; EGFR) family, with antineoplastic activity. Afatinib more effectively sensitizes lung cancer cells (Lewis lung carcinoma cells) to radiation and decreases metastasis by inhibiting phosphorylation of EGFR and HER2 and partly by decreasing matrix metalloproteinase 9 (MMP-9) production (56). Cancer cell resistance to chemotherapy and radiotherapy through EGFR overexpression negatively affects therapeutic success (56, 58). Expression of the wild-type EGFR in glioma cancer cells (F98_EGFR_) and its morbid mutant (F98_EGFRvIII_) isoform has contributed to the development of novel targeted dual therapy in combination of anti-EGFR drugs with BNCT. On the one hand, anti-cancer compounds containing a BPA conjugate with an epidermal growth factor (EGF) ligand or anti-EGFR antibody (mAbs, cetuximab (C225)) or anti-EGFRvIII mutation antibody (L8A4), and specifically recognize wild-type EGFR and EGFRvIII. However, they selectively deliver large amounts of boron to gliomas necessary for BNCT. *In vivo* studies in rats bearing complex tumors (F98_EGFR_/F98_EGFRvIII_) have shown that it is necessary to target BPA to cells expressing both EGFR and EGFRvIII to homogeneously distribute boron in gliomas, enabling the breakthrough therapeutic effects of BNCT (59). This type of dual therapy reduces the chemotherapeutic and radiological resistance in cancer cells.

Only a limited number of *in vitro* studies during BNCT have been undertaken regarding the heterogeneity of the tumor microenvironment including hypoxia, cancer stem cells, low blood flow, or low nutrition (60). These factors can cause tumor cells to become quiescent (Q), reducing radiosensitivity or inhibiting drug entrance, making the tumor more resistant to the treatment and causing recurrence. Oxygenated Q tumor cells have a greater ability to recover from DNA damage after anti-cancer therapy and suggest an interrelationship with CSCs (60, 61). CSCs are a subpopulation of cells within a tumor with stem cell-like properties (62). This population is considered to be resistant to conventional radiotherapies and chemotherapies, and is widely omitted in *in vitro* BNCT studies. However, recent research was performed using glioma stem-like cells (GSC), subpopulation of glioma cells, responsible for the stemness, quiescence, and therapy resistance, maintained by GSC niches in the microenvironment of the tumor (63). This study aimed to investigate BPA uptake by GSCs using flow cytometry (*in vitro*) and a mouse orthotopic tumor model (*in vivo*) and demonstrated that BNCT can target the destruction of GSCs and be an efficient therapy for malignant gliomas. Including this population in *in vitro* studies will further enhance the therapeutic properties of BNCT. Since glioblastoma (GBM) is the most lethal primary brain tumor and finding novel effective combined therapies is an urgent issue, we propose a useful model system for glioblastoma cell lines, M059K and M059J, to study the role of DNA protein kinase in cellular and molecular processes involving DNA damage recognition and repair. Based on the description in American Type Culture Collection (ATCC), M059K cells express normal levels of DNA-PKcs from the NHEJ pathway, whereas M059J cells lack DNA-dependent protein kinase activity. M059K cells are approximately 30-fold less sensitive to ionizing radiation than M059J cells. This model system could be used to study the kinetics of DNA repair after BNCT, similar to the study on γ-radiation (64). Detailed evaluation of repair pathways and the response to ionizing radiation in different cell subpopulations, including tumor microenvironment and niches, is essential to elucidate the molecular mechanisms of radiation-induced DNA damage and repair. Further understanding of BNCT-induced DDR mechanisms will lead to improved therapeutic efficiency against different tumors.

## Discussion

DNA-DSB repair is a composite process that relies on different factors, including DSB-inducing agents, cell cycle phase, cell cycle checkpoints, ncRNAs, and gene mutations in different cancer cell lines (36, 53). Based on many studies, it is known that the presence and quality of radiation-induced DSBs depend on the density of radiation, which should potentially have a significant impact on the choice of the repair pathway. In the present study, we pointed out that the molecular mechanisms activated by BNCT are poorly established, with no clear conclusions, prompting us to describe DDR by comparing the repair mechanisms in different cell lines. The NHEJ, in comparison with HRR, acts as an effective repair pathway across the entire cell cycle. Phosphorylated DNA-PKcs involved in this pathway plays a crucial role by binding to the DNA ends, and thus, making a choice between the two pathways. Moreover, DNA-PKcs is only recruited to longer-lived complex DSBs and could play an essential role in repair after the BNCT mixed radiation field (26, 34).

Understanding the molecular mechanisms of the effects of mixed radiation causing both complex and simple DSBs is important from the point of view of radiation protection and future design of combined radiotherapies, including the side effects and secondary emitted γ-rays. It is unclear how mammalian cells react after exposure to BNCT mixed radiation field, and how cells preferentially select a specific pathway to repair DSBs generated by high LET radiation. Do these cells express the highest levels of genes encoding proteins in DDR pathways? Are cells mainly focused on repairing simple DSBs leaving the repair of complex DNA damage? Finally, how can small ncRNAs regulate DDR during BNCT? These questions remain unanswered.

## Author Contributions

Conceptualization: KM-O; manuscript writing: KM-O, DK, MA, KT, and AK; figures: KM-O; supervision: AK. All authors contributed to the article and approved the submitted version.

## Funding

KM-O was supported by the National Science Centre, Poland (Miniatura 2), grant no. #2018/02/X/NZ5/02849. DK was supported by the project National Science Centre, Poland (ETIUDA 8), grant no. #2020/36/T/ST4/00485. The conducted research was partially financed by the Plenipotentiary Representative of the Government of the Republic of Poland at JINR in Dubna as part of the PWB/168-36/2021 Cooperation Project.

## Conflict of Interest

The authors declare that the research was conducted in the absence of any commercial or financial relationships that could be construed as a potential conflict of interest.
